# Selection, Characterization and Application of Nucleic Acid Aptamers for the Capture and Detection of Human Norovirus Strains

**DOI:** 10.1371/journal.pone.0106805

**Published:** 2014-09-05

**Authors:** Blanca I. Escudero-Abarca, Soo Hwan Suh, Matthew D. Moore, Hari P. Dwivedi, Lee-Ann Jaykus

**Affiliations:** Department of Food, Bioprocessing and Nutrition Sciences, North Carolina State University, Raleigh, North Carolina, United States of America; University of Houston, United States of America

## Abstract

Human noroviruses (HuNoV) are the leading cause of acute viral gastroenteritis and an important cause of foodborne disease. Despite their public health significance, routine detection of HuNoV in community settings, or food and environmental samples, is limited, and there is a need to develop alternative HuNoV diagnostic reagents to complement existing ones. The purpose of this study was to select and characterize single-stranded (ss)DNA aptamers with binding affinity to HuNoV. The utility of these aptamers was demonstrated in their use for capture and detection of HuNoV in outbreak-derived fecal samples and a representative food matrix. SELEX (Systematic Evolution of Ligands by EXponential enrichment) was used to isolate ssDNA aptamer sequences with broad reactivity to the prototype GII.2 HuNoV strain, Snow Mountain Virus (SMV). Four aptamer candidates (designated 19, 21, 25 and 26) were identified and screened for binding affinity to 14 different virus-like particles (VLPs) corresponding to various GI and GII HuNoV strains using an Enzyme-Linked Aptamer Sorbant Assay (ELASA). Collectively, aptamers 21 and 25 showed affinity to 13 of the 14 VLPs tested, with strongest binding to GII.2 (SMV) and GII.4 VLPs. Aptamer 25 was chosen for further study. Its binding affinity to SMV-VLPs was equivalent to that of a commercial antibody within a range of 1 to 5 µg/ml. Aptamer 25 also showed binding to representative HuNoV strains present in stool specimens obtained from naturally infected individuals. Lastly, an aptamer magnetic capture (AMC) method using aptamer 25 coupled with RT-qPCR was developed for recovery and detection of HuNoV in artificially contaminated lettuce. The capture efficiency of the AMC was 2.5–36% with an assay detection limit of 10 RNA copies per lettuce sample. These ssDNA aptamer candidates show promise as broadly reactive reagents for use in HuNoV capture and detection assays in various sample types.

## Introduction

Human noroviruses (HuNoV) are the most common cause of acute viral gastroenteritis worldwide [1]. Members of the *Caliciviridae* family, these viruses are transmitted by a variety of routes, but frequently cause outbreaks in closed settings such as schools, nursing homes, and cruise ships. Contamination of foods and water is another common transmission mode, as HuNoV are the leading cause of foodborne disease in the U.S. [2] and perhaps worldwide [3] [4]. Low infectious dose, high virus concentrations in the feces and vomitus of infected individuals, lengthy environmental persistence, and resistance to many commonly used sanitizers and disinfectants all contribute to the high degree of transmissibility of HuNoV [5].

Despite their public health significance, routine detection of HuNoV in community settings or in food and environmental samples, is limited. Firstly, there is no cell culture model to propagate these viruses. Secondly, HuNoV have tremendous antigenic diversity which has complicated the development of broadly reactive antibodies, meaning that enzyme immunoassays have poor sensitivity [6] [7]. While molecular amplification methods (specifically reverse transcriptase quantitative PCR or RT-qPCR) are commonly used in public health settings, these methods are rarely used in clinical diagnostic laboratories in the U.S. Detection of HuNoV in food and environmental samples is even more complicated because virus concentrations are so low in these samples that it is necessary to perform labor intensive and relatively inefficient pre-concentration step(s) prior to detection [8].

There is a need to develop alternative HuNoV diagnostic reagents to complement existing ones. Nucleic acid aptamers are short ssDNA or ssRNA sequences having binding affinity for a target molecule, like bacteria, viruses, or cells. Once identified, they offer advantages over other binding ligands such as ease of production, regeneration and stability [9]. From a diagnostic perspective, they have been used for both target capture and detection purposes [9]. In this study, we describe the selection and characterization of ssDNA aptamers with binding affinity to HuNoV. The utility of these aptamers was demonstrated in their use for capture and detection of HuNoV in outbreak-derived stool samples and a representative food matrix.

## Materials and Methods

### Viruses and Virus-Like Particles (VLPs)

#### Viruses

Snow Mountain virus (SMV), the prototype genogroup II, genotype 2 (GII.2) HuNoV and the target for aptamer selection, and Norwalk (NV) the prototype genogroup I, genotype 1 (GI.1) strain were obtained as stool specimens originating from a human challenge study (courtesy of C. L. Moe, Emory University, Atlanta, GA). The SMV human challenge study was conducted at the University of North Carolina at Chapel Hill (UNC-CH) and was approved by the UNC-CH Biomedical IRB. The NV study was conducted at Emory University and approved by the Emory University IRB, Biomedical Committee. Both studies had written consent, with each participant signing an informed consent witnessed by trained study staff. The informed consent documents were approved by the IRBs. The Emory University group also supplied pre-challenge stool samples confirmed (by RT-qPCR) as negative for HuNoV. These were used for counter selection and as negative controls in some studies. Additional fecal specimens associated with previously confirmed HuNoV outbreaks (representing strains GI.6, GII.1, GII.3, GII.4, GII.7 and an untypable GII) were also used in detection assays (courtesy of S.R. Greene, North Carolina Department of Health and Human Services, Raleigh, NC). All stool samples were suspended 20% in phosphate buffered saline (PBS). In some cases, these suspensions were used without further purification, designated as crude suspensions. In other cases, the suspensions were partially purified using chloroform extraction [10]. Hepatitis A virus HM175 (cell culture adapted) and poliovirus 1, routinely used in our laboratory, were partially purified (chloroform extracted) from cell culture lysates and used in exclusivity studies. All virus suspensions were stored at −80°C until use.

#### Virus-Like Particles (VLPs)

Self-assembled non-infectious Virus-Like Particles (VLPs), produced using the recombinant expression of HuNoV capsid proteins were used as purified candidate proteins for the characterization of aptamer binding affinity. The VLP panel consisted of representatives of genogroup I [GI.1 (Norwalk virus), GI.4, GI.6, GI.7 and GI.8)] and genogroup II [GII.1, GII.2 (SMV), GII.3, GII.4 (Houston and Grimsby virus), GII.6, GII.7, GII.12 and GII.17] HuNoV (kindly provided by R. Atmar, Baylor College of Medicine, Houston, TX). VLPs were stored at 4°C until use.

### Selection of Aptamers using SELEX (Systematic Evolution of Ligands by EXponential Enrichment)

#### Preparation of the DNA Library

All primer and probe sequences used in this study were obtained from Integrated DNA Technologies (Coralville, IA). The 81-base combinatorial DNA library consisted of 40-mer random regions flanked by forward and reverse constant regions [5′-AGTATACGTATTACCTGCAGC-(N)_40_-CGATATCTCGGAGATCTTGC-3′]. Preparation of the dsDNA library for SELEX was done in accordance with the method of Dwivedi et al. (2010) [11]. Specifically, the library was amplified using a fluorescein (FAM)-labeled forward constant region primer [5′-56-FAM/-AGTATACGTATTACCTGCAGC-3′] and a biotinylated reverse constant region primer [5′-/5Bios/-GCAAGATCTCCGAGATATCG-3′]. Briefly, a 50 µl reaction master mix containing 5 µl of aptamer library (10 µM), 1x GoTaq buffer (Promega Corp., Madison, WI), 0.2 mM dNTPmix (Applied Biosystems, Foster City, CA), 5 U Go Taq DNA polymerase (Promega), and 500 nM of each primer were amplified as follows: 95°C for 5 min, followed by 30 cycles of 95°C (1 min), 55° (1 min), 72°C (1 min) and a final extension at 72°C for 10 min, using a DNA Engine (PTC-200) Peltier Thermal Cycler-200 (MJ Research/Bio-Rad Laboratories, Hercules, CA). The labeled dsDNA library was conjugated to Streptavidin MagneSphere Paramagnetic particles (SA-PMPs) (Promega) according to manufacturer instructions and harvested with an MPC-M magnetic particle concentrator (Invitrogen Dynal AS, Oslo, Norway). The library-magnetic bead conjugate was washed three times in 0.1X SSC. The FAM-labeled ssDNA moieties were then separated from the immobilized biotinylated strands by alkaline denaturation using 50 µl of 0.15 M NaOH for 7 min at room temperature (RT), followed by magnetic separation of the beads. The supernatant obtained was mixed with 1 ml of nuclease free water followed by filtration to remove residual NaOH using a YM-30 filter device (Millipore, Billerica, MA). It was then purified by ethanol precipitation with resuspension in 50 µl of nuclease free water. The presence of FAM-labeled DNA was confirmed using a fluorescent plate reader (Tecan Safire, Tecan Group Ltd., Männedorf, Switzerland) and its concentration determined using a NanoPhotometer Pearl (Implen GMbH, Munich, Germany).

#### Preparation of the target for SELEX

The target for SELEX was produced by immobilizing SMV to antibody-bead conjugates. Briefly, M-280 Tosylactivated Dynabeads (Invitrogen Dynal) were cross-linked to mouse monoclonal antibodies against SMV (Abcam, Cambridge, MA) as per manufacturer instructions. One hundred µl of the partially purified SMV stock (consisting of approximately 10^5^–10^6^ RT-qPCR amplifiable units/ml) was mixed with 10 µl of the antibody-bead conjugate suspended in 500 µl of binding buffer [consisting of phosphate buffered saline (pH 7.1) supplemented with 100 mg/L CaCl_2_, 100 mg/L MgCl_2_ and 0.05% Tween 20] followed by RT incubation for 2 h. After washing thrice with PBS supplemented with 0.05% Tween 20 (PBST), the conjugate was resuspended in 20 µl of PBS and used for SELEX.

#### SELEX

An aliquot of 300–500 pmoles of FAM-ssDNA pool suspended in 500 µl of binding buffer was added to 10 µl immobilized SMV target followed by gentle rotation for 45 min at RT. Aptamer-bound SMV was recovered by magnetic capture, washed thrice in PBST, and the aptamers eluted from the bead-bound virus particles with 200 µl of nuclease free water followed by heating at 90°C for 5 min. The resulting aptamer pool was purified by ethanol precipitation, re-amplified using the labeled constant region primers, and the FAM-ssDNA aptamers were recovered as described above. This constituted one selection round and a total of nine such iterations of the selection process were performed. To avoid non-specific amplification of the recovered aptamer pool, single stranded binding protein (Promega) at a concentration of 0.1 µg/µl was added to the PCR reactions and the pool was amplified using the appropriate annealing temperatures (obtained by running a temperature gradient within a range of 55°C to 65°C on the recovered aptamer pool).

#### Counter-SELEX

Two sequential counter-SELEX rounds were done after each round of SELEX round to impart specificity to the aptamer candidates against (1) the components of a 20% HuNoV-negative human stool suspension; and (2) the bead-antibody complex (without SMV). In counter-SELEX, exposure of the aptamer pools to the negative stool specimens or bead-antibody complex was done as described above, but in this case, the aptamers bound to the complex were discarded, while the unbound aptamer pool (supernatant) was recovered. This was purified by ethanol precipitation, re-amplified by PCR, and used in another round of SELEX.

#### Identification of Aptamer Sequences

After the 4^th^, 7^th^, and 9^th^ rounds of sequential SELEX and counter-SELEX, final PCR product was cleaned using the QIAquick PCR purification kit (Qiagen, Valencia, CA) and cloned into TOPO vector (Invitrogen, Carlsbad, CA) according to manufacturer instructions. The DNA insertion in each clone was sequenced by Genewiz (South Plainfield, NJ). The unique aptamers were identified by sequence analysis.

### Characterization of Aptamer Candidates

#### Dissociation Constant and Structural Analysis of Aptamers

Consistent with the approach of others [12] [13], an ELISA-like assay (Enzyme-Linked Aptamer Sorbant Assay, or ELASA, described below) was used to determine equilibrium dissociation constants (K_d_) for the candidate aptamers. This was done using SMV VLPs (3 µg/ml) and different concentrations (10 nM, 100 nM, 500 nM, 1 µM, 2 µM) of each aptamer. To estimate K_d_, plots of the ratio between absorbance of Test samples/absorbance of Negative controls (T/N ratios, Y axis) as a function of the aptamer concentration (X axis) were generated using a non-interacting binding sites model in Sigma Plot (Jandel, San Rafael, CA). Common sequence motifs were identified using the online MEME server (http://meme.sdsc.edu). Structural folding analyses of the selected aptamers were done using the DNA Mfold online server (http://mfold.rna.albany.edu/?q=mfold/DNA-Folding-Form) [14]. To identify potential binding epitopes, the major capsid protein (VP1) sequences of each of the VLPs tested were aligned using Clustal W alignment in the Molecular Evolutionary Genetics Analysis program (MEGA 6.0) (http://www.megasoftware.net/). Aligned sequences were matched with the aptamers that showed the strongest (+++) binding affinity to each VLP.

#### Binding Affinity Studies Using Enzyme-Linked Aptamer Sorbent Assay (ELASA)

Binding affinity studies were performed with candidate aptamers and VLPs using a protocol adapted from a previously reported ELISA-based antigen detection assay [15]. In this case, the antibody was replaced with an aptamer and the resulting procedure termed Enzyme-Linked Aptamer Sorbent Assay (ELASA). For this assay, the selected aptamers were labeled with a 5′ biotin moiety. One hundred µl of pure VLP suspension (3 µg/ml) was placed in each well of a covered, flat-bottomed polystyrene 96-well plate (Costar 3591, Fisher, Pittsburg, PA) and incubated overnight at 4°C. After coating the wells with VLPs, the plates were blocked with 200 µl of 5% skim milk in PBST containing non-related DNA sequences (i.e., *L. monocytogenes* primers hlyQF/R and L23SQF/R) [16], followed by overnight incubation at 4°C. After washing three times with PBST, 100 µl of biotinylated aptamer (1 µM) was added to each well and the plate was incubated for an hour at RT with gentle mixing. After removing excess aptamers, the plates were washed three times with PBST. One-hundred µl of ELISA-grade streptavidin-horse radish peroxidase conjugate (1 mg/ml, 1∶5000, Invitrogen) was added to the plate and incubated at RT for 15 min. The unbound conjugate was removed and the plate was washed five times with PBST before applying 100 µl of 3,3′5,5′tetramethylbenzidine (TMB) peroxidase substrate (KPL, Gaithersburg, MD) to each well. The plate was incubated for 5 min at RT after which 100 µl of 1 M phosphoric acid was added to stop the reaction. Absorbance at 450 nm was recorded using a microplate reader (Tecan Infiniti M200pro). Negative controls consisted of no VLPs. As per convention [17], binding affinity was interpreted based on the ratio between the absorbance readings for the test samples (to which labeled aptamer had been added) versus those for the negative control (PBS alone), (T/N ratios). Ratios ≤2.0 were considered negative [17]. Ratios between 2.0 and 5.0; >5.0 and 10.0; and >10.0 were interpreted as low, medium or strong binding, respectively. Ratios obtained for the negative control were in the range of 0.1–0.3. To evaluate binding inclusivity, ELASA was performed using 1 µM (100 µl) of each candidate aptamer as applied to a panel of virus-like particles (VLPs) corresponding to genogroup I [GI.1 (Norwalk virus), GI.4, GI.6, GI.7 and GI.8)] and genogroup II [GII.1, GII.2 (SMV), GII.3, GII.4 (Houston and Grimsby), GII.6, GII.7, GII.12 and GII.17] HuNoV. Exclusivity analyses were done by ELASA using hepatitis A virus (HAV) and poliovirus. A scrambled random sequence for aptamer 25 (25 S) was generated by the Random Nucleic Acid Sequence Generator server on line http://molbiol.ru/eng/scripts/01_16.html and used to evaluate background binding in the ELASA. In all cases, PBS and SMV-VLPs were included as negative and positive controls, respectively.

#### Binding affinity Studies Using Enzyme-Linked Immunosorbent Assay (ELISA)

The original ELISA protocol adapted for ELASA was used in these experiments [15]. Briefly, SMV-VLPs were used to coat 96-well polystyrene plates as described above. The wells were blocked with 200 µl of 5% skim milk in PBST and incubated for 2 h at RT. After washing 3 times with PBST, 100 µl of diluted (1∶5000) GII.2 antibody (Abcam, Cambridge, MA) was added and incubated for 1 h at 37°C. After washing 3 times with PBST, 100 µl of the secondary antibody (anti-mouse conjugated with horseradish peroxidase; Abcam, Cambridge, MA) at a 1∶5000 dilution was added to each well and incubated for 1 h at 37°C. The plate was washed 3 times with PBST with subsequent development using TMB peroxidase substrate and absorbance reading was recorded as described above for ELASA.

### Application of Aptamers for Detection of HuNoV in Clinical and Food Samples

#### Detection of HuNoV in Outbreak-Derived Stool Samples

The ELASA assay was used to assess the performance of select aptamer candidates for detection of HuNoV in outbreak-derived fecal suspensions. Specimens evaluated included those representing strains GI.1 (Norwalk), GI.6, GII.1, GII.2 (SMV), GII.3, GII.4, GII.7 and an untypable GII. Briefly, 100 µl of ten-fold serially diluted partially purified fecal suspensions were used to coat plates followed by incubation overnight at 4°C. After three washes with PBST, the ELASA assay was done as described above. PBS alone and fecal suspensions derived from uninfected individuals were used as negative controls. Due to limited availability of SMV VLPs, we used GII.4 (Houston) VLPs, which were also highly reactive to aptamer 25, as the positive control.

#### Detection of HuNoV in Artificially Contaminated Lettuce Samples Using a Combined Pre-Concentration-Aptamer Magnetic Capture (AMC)-RT-qPCR Assay

For virus inoculation and pre-concentration, 3 g of lettuce (approximately 3×3 cm square) was disinfected by UV light and inoculated with 200 µl of serially diluted crude GII.4 virus stock suspension (due to limited availability of SMV) at inoculum concentrations ranging from 1–5 log_10_ RNA copies per lettuce sample. The inoculum was allowed to dry for 30 min. Virus pre-concentration was done using a previously reported elution-concentration method [18]. Briefly, the samples were mixed with 25 ml of 0.5 M glycine-0.14 M NaCl buffer (pH 9.0), placed in sterile Whirl-Pak-filter bags (Nasco, Fort Atkinson, WI) and stomached (Stomacher 400 Circulator, Seward, Norfolk, UK) for one min. The recovered filtrate (containing the eluted viruses) was adjusted to 0.9 M NaCl and supplemented with 1% bovine serum albumin (Sigma Aldrich, St. Louis, MO), after which 12% polyethylene glycol (PEG) MW 8,000 (Sigma Aldrich) was added. After incubation for 2 h at 4°C, samples were centrifuged at 10,000×*g* for 20 min at 8°C and the recovered pellet was resuspended in 1 ml of PBST.

For aptamer magnetic capture (AMC), the resuspended pellet was incubated with 1 µM of biotinylated aptamer for an hour at RT with rotation. This was followed by the addition of 10 µl of Streptavidin (SA)-C1 magnetic beads (from 10 mg/ml stock solution) (Invitrogen Dynal AS, Oslo, Norway) previously blocked overnight with 5% skim milk in PBST followed by incubation for 25 min at RT. The bead-aptamer-virus conjugates were recovered using the magnetic particle concentrator. The conjugates were washed twice with PBST, suspended in 100 µl PBS, and the RNA extracted using the easyMAG automated system (bioMerieux SA, Marcy l'Etoile, France) according to manufacturer's instructions. The viral RNA was eluted in 40 µl of proprietary elution buffer and stored at −80°C until used for amplification.

RT-qPCR was carried out using the Superscript III Platinum One-Step RT-qPCR system (Invitrogen) according to manufacturer instructions. Specifically, 25 µl RT-qPCR reactions consisted of 12.5 µl of 2X reaction mix, 5.5 µl DNase-RNase free water, 200 nM of each primer [JJV2F (5′-CAAGAGTCAATGTTTAGGTGGATGAG-3′) and COG2R (5′-TCGACGCCATCTTCATTCACA-3′)], and probe RING2-TP [5′-56-FAM TGGGAGGGCGATCGCAATCT-1BHQ-3′] [19] [20], 0.5 µl of the enzyme mix (SuperScript III RT/Platinum Taq Mix) and 5 µl of the RNA template. Amplification was done under the following conditions: 50°C for 15 min, 95°C for 2 min followed by 45 cycles of 95°C for 15 s, 54°C for 30 s, and 72°C for 30 s in a SmartCycler (Cepheid, Sunnyvale, CA). The RNA copy number was extrapolated from a standard curve based on Ct values obtained by RT-qPCR amplification of serially diluted synthetic RNA as previously reported [21]. Capture efficiency, expressed as a percentage, was estimated from the standard curve and calculated as the ratio of the extrapolated RNA copies (after capture and detection by RT-qPCR) to the total input RNA copies per sample, multiplied by 100 [22]. Negative controls consisted of capture by blocked beads in the absence of aptamer.

### Statistical analysis

Data were expressed as mean ± standard deviation of three replicates of each experiment. The data were analyzed by one-way analysis of variance (ANOVA) with the Tukey's multiple comparison test using GraphPad Prism ver. 5.0d (San Diego, CA). Values of *p*<0.05 were considered statistically significant.

## Results

### ssDNA Aptamer Selection

After 4, 7, and 9 rounds of sequential SELEX and counter-SELEX, 34 unique aptamer candidates were identified from a total of 80 clones sequenced. All sequences are provided in Table 1. Candidates designated as 19, 21, 25 and 26 were selected for further analysis as they were the most abundant in the identified aptamer pool (identified between 5–10 times) and showed strong preliminary binding affinity for SMV, genogroup I.1 (Norwalk), and genogroup II.4 (Houston) VLPs using the ELASA assay.

**Table 1:** Aptamer sequences obtained from 4^th^, 7^th^ and 9^th^ round of SELEX for SMV.

ROUND	RANDOM REGION SEQUENCE	# OF REPEATS	APTAMER SELECTED
OF SELEX		IN THE POOL	WITH CODING
4	TGTTGGATTTTACGAAAAACGTGCTTACTTCATAGCGGCC	1	
4	GGTTGGGTAAGGGGGTCTGGTCAGGTAGGGCGGGGGGGGG	1	
4	TCGTAAACCCCTTATCCGTGAACCTTCAGCGGTAGACGCT	2	
4	CTCCCTCCAGCCTGCCTATTTTGCTTGGTTACGCATCTGT	1	
7	CCAGCGAAGGAAAGTCTTGGTTGGTCTAGTTTTTCGTGTG	2	
7	CTACGTGTGCGTTCCGATTGTTTAAATTGCTCAATGTATG	2	
7	CACACCACCTGAATTCCAGCACACTGGCGGCCGTTACTAG	2	
9	CACTCGACCTTCAGGGCGGCTTCTCAGCGTGTAGTGGTGA	1	
9	CTCGACTGATAGACCTAGCGTCAATCCTCATTGTTCGCTG	1	
9	CCAGTATTAGAGTCCTACTTTACACCGCTCTTGGCATCGT	1	
9	CACATGATAAGGTCGCGTGACTGTGAGTTAGTTGTTACAC	2	
9	TCGGCATAGGTCAAGTCGCTTCATTTGGATTAAGTTGAGG	1	
9	CACATACCAAAGTATTGGTCGCTAACTTTCGCCCAATTGA	1	
9	CTACGAGGTGGTTATAAGAGAACTTATCCGTGTTGGTTGC	1	
9	TGGTAGTGGGATATAGTTTTTCCAAGCGTACCCAGTTCTG	2	
9	CTATCAGCCATGAATTGCATTACCTTTGTTCTCCCCTTGC	1	
9	CCCCTCGGAAGATAGATTTTGCGAGAGTCTTGGGTTGAGG	1	
9	CCAGATAGCAGCACCTAATCTTATCCCTTTTATTTTTGGT	2	
9	TCGGGGGGAGGAGGGGGAATGGGAAGAAGGAGGTCGAGGG	1	
9	TGGATTACACGGCTAACTTCCCTGGTTCTTTTCTTTGATG	2	
9	TGGACGTTATTTGCACTCGTCGAACCCTATCATGCCTCCT	1	
9	CCTCATGCACAAAGGCTTATTACGGTCTAATTCTTTATAA	1	
9	TCGACATTATGTTTGACATCGATTGTTAATGTTTCTTTGC	2	
9	CCCCTACACAGTAAAATTCTTTAACACCTAGATCTTCGAC	2	
7, 9	**CACCAGTGTGTTGAGGTTTGAGCACACTGATAGAGTGTCA**	9	SMV-19
9	TGAGCCTCCGTTTTAGTGATCAGAAGGGATGTGTGGCGTA	1	
9	**CCATGTTTTGTAGGTGTAATAGGTCATGTTAGGGTTTCTG**	9	SMV-21
9	CGAGGGATACATGCTGACTATGGAATTATTTGAATTCCCA	4	
9	CTACAGGAGTTCATCTGGGAGAGTGTAAAGGATGAGGTGG	2	
7, 9	**CATCTGTGTGAAGACTATATGGCGCTCACATATTTCTTTC**	10	SMV-25
9	**TGACCGAGTGTCTGGTCATTTTCGATGTCTGTTGTTAGGC**	7	SMV-26
9	CCCTCCTTATCTCTGCTAATGGTTGATCCGTGTCCCGTAC	1	
9	CCCTGTTATCCTTATCCAACGAGCTTAATGTAACTTGGAC	2	
9	TGGGGGAGTGGTAGGTGTGCTGTGAAGGGGAGGGTTGGGG	1	

Bolded sequences were used in characterization studies and applications.

### Characterization of ssDNA Aptamer Candidates

**Figure 1:**
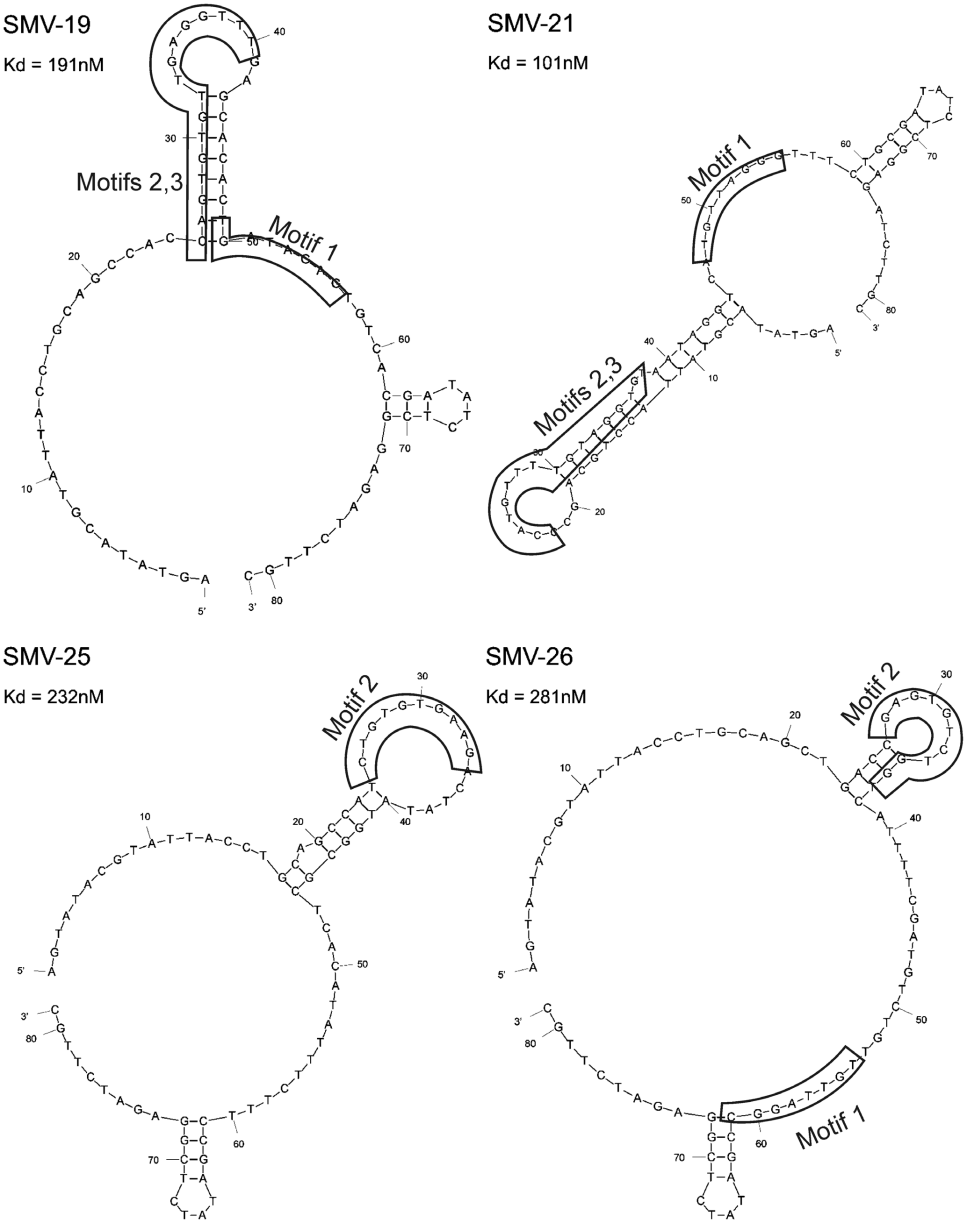
Predicted structural folding of select ssDNA aptamers for SMV. Common motifs identified in boxes.

**Figure 2:**
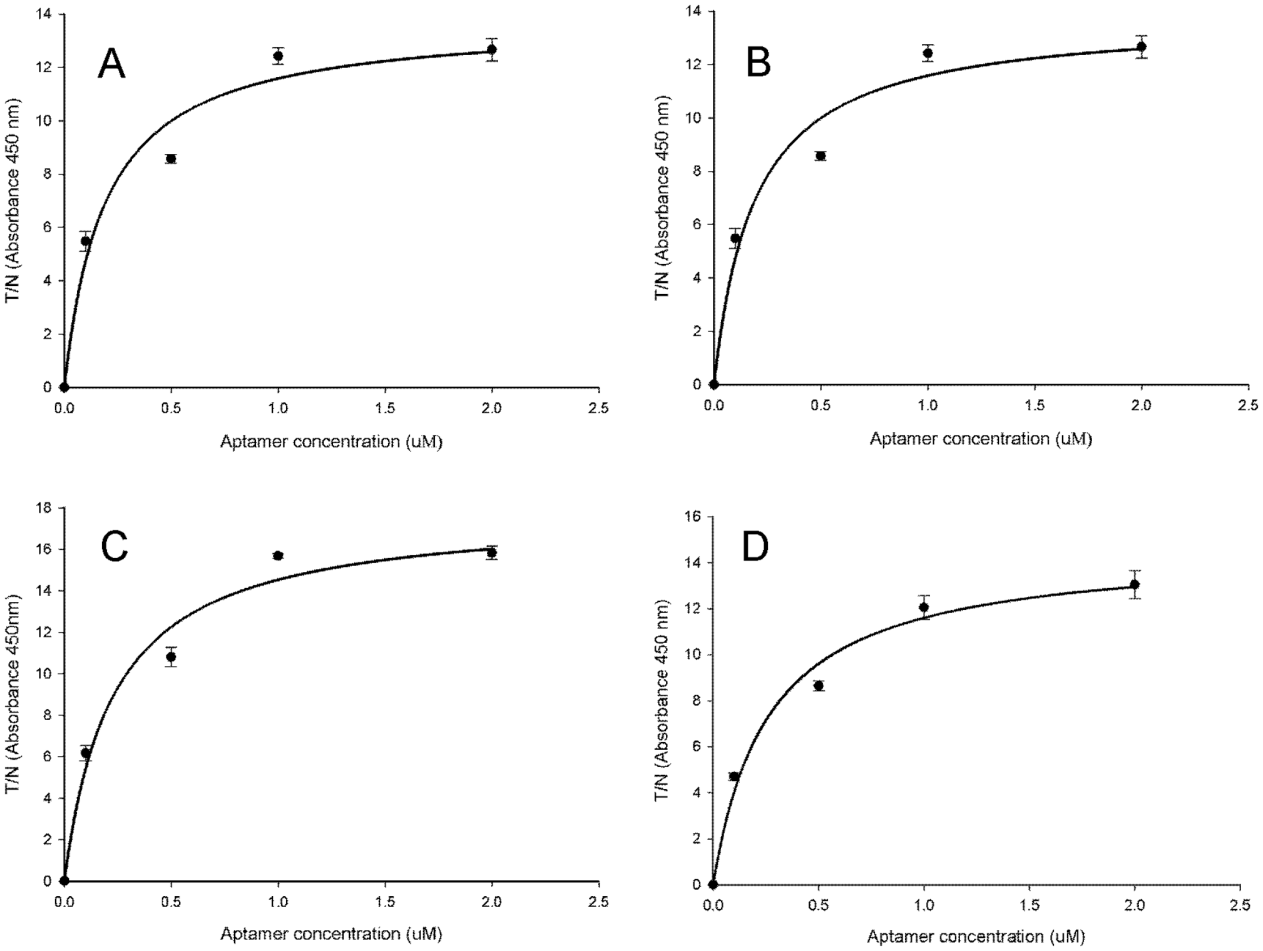
Equilibrium dissociation curves for select aptamers. GII.2. SMV VLPs (3 µg/ml) were screened with different concentrations (10 nM, 100 nM, 500 nM, 1 µM, 2 µM) of each aptamer using ELASA. To estimate K_d_, plots of the T/N ratios (absorbance at 450 nm) as a function of the aptamer concentration were fitted to a non-interacting binding sites model with the equation *Y* = Bmax *X*/Kd+*X*. The negative controls consisted of PBS. Results correspond to aptamers SMV 19 (A), SMV 21 (B), SMV 25 (C) and SMV 26 (D).

The structural folding for all four aptamers demonstrated a dominant loop and two protruding hairpins (Figure 1). Three motifs were observed when comparing the random sequence regions of the four aptamers. Motif 1 was found in aptamers 19, 21, and 26. Motif 2 was found in all four aptamers and was in most cases, involved in stem-loop formation. Motif 3, immediately downstream of motif 2, was found in aptamers 19 and 21 and was also involved in stem-loop structures. Equilibrium dissociation constant (K_d_) values approximated for the aptamers were 191 nM for aptamer 19; 101 nM for 21; 232 nM for 25; and 281 nM for 26. Figure 2 shows the corresponding K_d_ curves generated using the one site binding model. The regression coefficients (R^2^) associated with this model ranged from 0.95 to 0.99.

Signal intensity ratios (T/N) in ELASA as evaluated for aptamers 19, 21, 25 and 26 using a panel of VLPs ranged from a low of 1.3 to a high of 18.1 (Table 2). Aptamer 21 demonstrated medium to high binding affinity with VLPs corresponding to GI.7, GII.1, GII.2, GII.3, GII.4 (both VLPs), GII.7, GII.12, and GII.17 and T/N ratios ranging from 6.4 to 18.1. Aptamer 25 reacted positively with VLPs corresponding to GI.4, GI.8, GII.1, GII.2, GII.3, GII.4 (both VLPs), GII.6, and GII.7 (T/N ratios ranged from 5.4 to 12.4). Aptamers 19 and 26 were less broadly reactive. In general, T/N ratios were higher for GII VLPs than for GI VLPs. Although all four aptamers showed variable binding affinity to different genotypes in genogroups I and II, binding affinity was highest for the GII.2 VLP, which is represented by SMV, the target used for the aptamer selection. Binding affinity was also quite high for GII.4 Houston VLPs.

**Table 2:** Binding affinity of selected aptamers against a broad panel of HuNoV VLPs.

VLPs	Aptamers^1^							
Genogroup	19		21		25		26	
GI.1 (Norwalk)	5.4±0.9	(+)	2.4±0.1	(+/−)	3.0±0.4	(+/−)	2.6±0.5	(+/−)
GI.4	1.7±0.3	(−)	4.3±0.2	(+/−)	5.6±0.2	(+)	1.1±0.2	(−)
GI.6	3.7±1.3	(+/−)	3.8±0.1	(+/−)	2.4±0.1	(+/−)	1.5±0.4	(−)
GI.7	10.3±0.6	(++)	8.1±0.1	(+)	3.5±0.4	(+/−)	1.8±0.8	(−)
GI.8	1.7±0.2	(−)	4.6±0.3	(+/−)	6.2±0.1	(+)	1.2±0.1	(−)
GII.1	7.1±0.3	(+)	8.6±0.3	(+)	9.4±0.1	(+)	2.1±0.3	(+/−)
GII.2 (SMV)	12.9±5.1	(++)	18.1±3.2	(++)	12.4±1.1	(++)	4.1±0.8	(+/−)
GII.3	1.7±0.9	(−)	11.8±2.7	(++)	5.4±0.1	(+)	2.4±0.5	(+/−)
GII.4 (Grimsby)	9.6±4.8	(+)	6.4±1.9	(+)	10.4±0.8	(++)	3.2±0.3	(+/−)
GII.4 (Houston)	12.5±4.2	(++)	11.3±1.2	(++)	11.0±1.1	(++)	2.8±0.4	(+/−)
GII.6	1.8±1.0	(−)	3.0±0.2	(+/−)	7.3±0.7	(+)	2.8±0.3	(+/−)
GII.7	4.0±1.9	(+/−)	13.5±1.4	(++)	9.1±2.1	(+)	2.8±0.8	(+/−)
GII.12	1.5±0.4	(−)	6.6±0.1	(+)	1.9±0.1	(−)	2.4±0.5	(+/−)
GII.17	3.0±1.6	(+/−)	12.2±0.3	(++)	2.0±0.1	(+/−)	1.3±0.1	(−)

1 Values indicate the ratio of absorbance readings for the test sample (T) versus negative control (N) using ELASA. Per convention [17], results less than 2.0 are considered negative (−). Low (+/−), medium (+) or strong (++) binding were interpreted for ratios between 2 and 5; >5 and 10; and >10, respectively. All experiments were done in triplicate.

**Figure 3:**
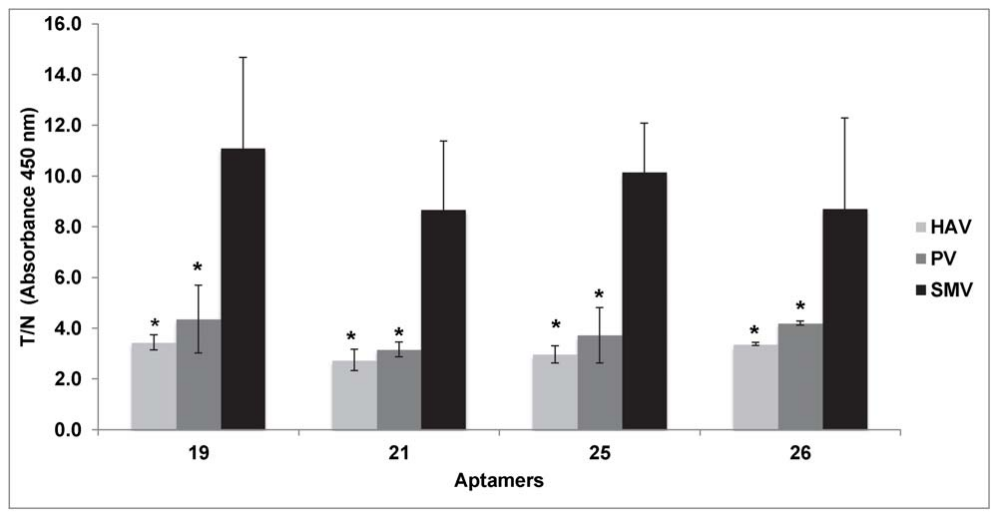
Binding of selected aptamers to hepatitis A virus (HAV) and poliovirus (PV). Cell culture lysates of poliovirus and HAV were tested using ELASA with candidate aptamers. Positive controls consisted of SMV-VLPs; negative controls consisted of PBS alone. Results are expressed as ratios between absorbance readings for test sample versus negative control (T/N). Experiments were done in triplicate. Statistically significant differences between the ratios obtained from the positive control and the samples are designated with an asterisk (p<0.05).

Relative to exclusivity analysis, binding affinity (T/N) of the four aptamers to the non-target virus poliovirus was in the range of 3.2–4.4, and for HAV, from 2.8–3.4. These values were statistically significantly lower (*p*<0.05) as compared to the positive control [GII.2 (SMV) VLPs] (Figure 3).

Aptamer 25 and a scrambled derivative (aptamer 25 S) were used in ELASA to demonstrate the potential for non-specific binding. The T/N ratios obtained by ELASA using GII.2 SMV VLPs and biotinylated aptamer 25 S were statistically significantly lower (*p*<0.05) when compared to those obtained using biotinylated aptamer 25. In competition studies, the binding of biotinylated aptamer 25 was not negatively impacted by the presence (in excess) of aptamer 25 S (*p*<0.05), demonstrating that non-specific binding of an irrelevant scrambled aptamer did not negatively impact the binding specificity of aptamer 25 to SMV VLPs (Figure S1).

**Figure 4:**
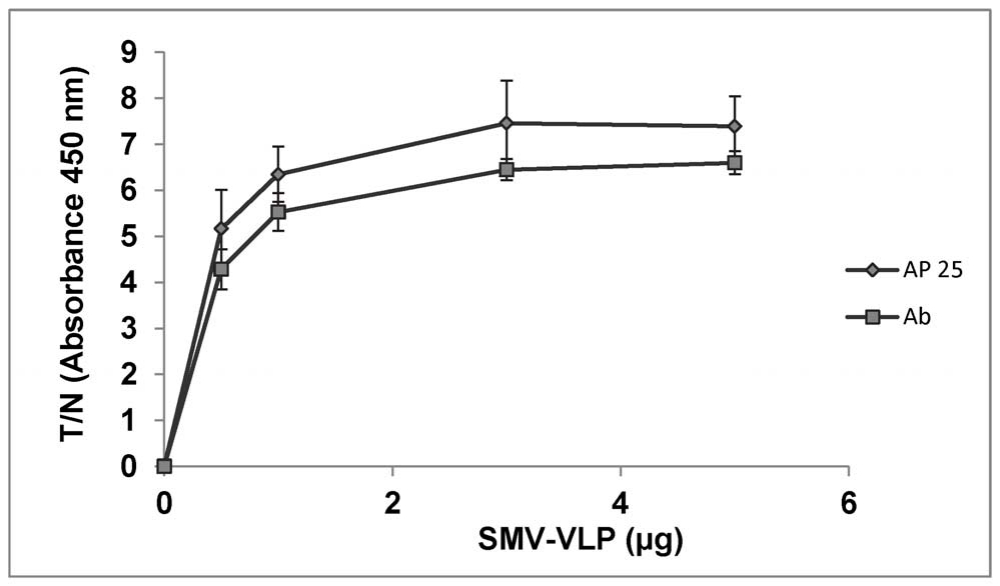
Comparison of ELASA and ELISA for detection of SMV-VPLs using aptamer 25. SMV-VLPs (1 to 5 µg) were suspended in partially purified 20% HuNoV-negative stool suspension, serially diluted, and detected using aptamer 25 (1 µM) (ELASA) or anti-GII.2 antibody (1∶5000) (ELISA). Negative controls consisted of PBS. Results are expressed as ratios between absorbance readings for test sample versus negative control (T/N). Experiments were done in triplicate. Statistically significant differences between the ratios obtained are designated with an asterisk (p<0.05).

For comparison purposes, ELASA and ELISA assays were performed using biotinylated aptamer 25 or a GII.2 commercial antibody, respectively, as applied to SMV VLPs suspended in partially purified HuNoV-negative stool. Based on comparison of T/N ratios, both assays performed equivalently within the aptamer/antibody concentration ranges of 1 to 5 µg/ml. (Figure 4).

### Application of Aptamers for Capture and Detection of HuNoV in Human Stool and Lettuce Samples

**Figure 5:**
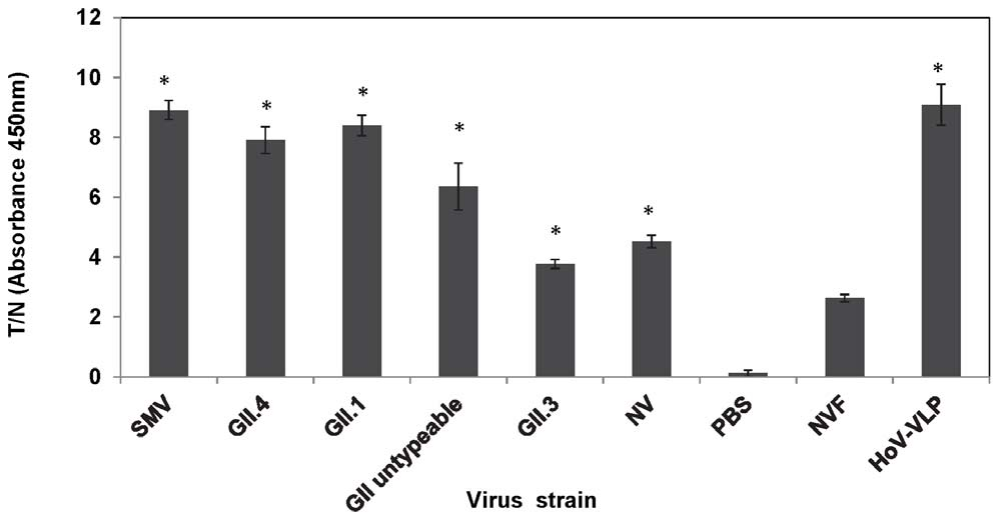
Binding of aptamer 25 to HuNoV stool specimens derived from outbreaks. Partially purified 10–20% stool suspensions were diluted and tested using ELASA. Negative controls consisted of PBS alone and HuNoV-negative human stool suspensions (NVF); the positive control was GII.4 (Houston) VLP. Results are expressed as ratios between absorbance readings for test sample versus negative control (T/N). Experiments were done in triplicate. Statistically significant differences between the ratios obtained from the positive stool specimens and the NVF are designated with an asterisk (*p*<0.05).

ELASA assays using aptamer 25 were performed on serially diluted partially purified outbreak-derived stool specimens. The T/N ratios corresponding to GI.1 (Norwalk), GII.1, GII.2 (SMV), GII.3, GII.4, and GII untypable outbreak specimens were all statistically significantly higher (*p*<0.05) when compared to the ratios obtained for either negative control samples (i.e., virus-free stool suspensions and no aptamer controls) (Figure 5). T/N ratios were higher for GII.2 (SMV), GII.1 and GII.4 outbreak specimens relative to GI.1 (Norwalk), GII.3, and GII untypable. Positive ELASA signals were not obtained for outbreak specimens corresponding to GI.6 and GII.7 HuNoV strains.

**Figure 6:**
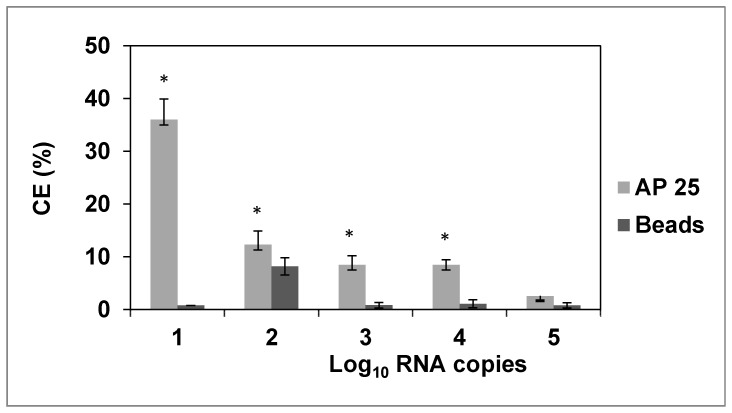
Performance of the AMC-RT-qPCR method (using aptamer SMV 25) as applied to artificially contaminated lettuce samples. Lettuce samples were inoculated with varying concentrations of a 20% suspension of HuNoV GII.4 fecal stock. They were pre-treated for virus concentration and purification using a combined elution-PEG precipitation method prior to AMC-qPCR. The negative controls consisted of the AMC using blocked beads in the absence of the aptamer. Experiments were done in triplicate. Statistically significant differences in recovery efficiencies are designated with an asterisk (*p*<0.05).

When virus on artificially contaminated lettuce samples (inoculated with GII.4 at levels ranging from 1–5 log_10_ RNA copies per sample) were pre-concentrated, used in AMC with aptamer 25, and detected after RNA extraction using RT-qPCR, a detection limit of about 1 log_10_ RNA copies per 3 g lettuce was obtained (Figure 6). Over this inoculum range, the capture efficiency of the combined pre-concentration-AMC-RT-qPCR assay ranged from 2.5–36%. Capture efficiency was significantly higher (p<0.05) than the negative controls which consisted of blocked beads in the absence of aptamer. Capture efficiency increased with decreasing virus concentration.

## Discussion

Nucleic acid aptamers are emerging molecules increasingly being used as diagnostic tools for detection of pathogens, including those transmitted by foodborne routes [9]. Two recent papers report on the development of aptamers directed against noroviruses [23] [24]. Specifically, Giamberardino et al. (2013) [23] produced a DNA aptamer against the cultivable surrogate murine norovirus (MNV) that displayed cross-reactivity to a GII.3 HuNoV VLP. This aptamer was used as a component of an electrochemical sensor that was able to detect as few as 180 MNV particles. Common to our approach, these investigators directed the SELEX process against the whole virus and used a similar number of selection rounds. Beier et al. (2014) [24] used a SELEX process directed against the capsid protein (VP1) of a GII.4 HuNoV to produce an aptamer for which they simulated the binding characteristics using predictive modeling. They did not evaluate the performance of the aptamer for virus capture or detection in clinical or food samples.

To our knowledge, the data presented here is the first time in which an investigative team has identified several different ssDNA aptamers that were extensively characterized for binding affinity using a wide range of HuNoV strains, both as VLPs and as intact viruses. This study also provides the first demonstration of the utility of aptamers in candidate capture-detection platforms, including those relevant to the fecal matrix and foods. Methodologically, it is novel because we employed extensive counter-SELEX to ameliorate potential non-specific binding. These features have led to some unique results.

Despite the targeting of a single virus (SMV) in SELEX, two of the identified aptamers (candidates 21 and 25) showed binding affinity to a panel of HuNoV VLPs, demonstrating the efficacy of SELEX in identifying aptamers with binding inclusivity to HuNoV strains. The binding inclusivity of aptamer 25 was further confirmed by the detection of HuNoV strains in outbreak-derived fecal samples by ELASA. The performance of aptamer 25 with outbreak stool specimens containing GII.1, GII.2, GII.3, and GII.4 HuNoV is consistent with the VLP binding data, all of which gave positive signals with ELASA. Likewise, poor performance of aptamer 25 with an outbreak specimen corresponding to GI.6 is consistent with the low degree of binding to that VLP. Binding of stool specimens containing GI.1 virus was inconsistent with VLP data, as was lack of binding to GII.7 specimens. Such inconsistency between VLP binding assays and application to outbreak-derived stool specimens could be a function of residual matrix associated aptamer binding (or interference with aptamer binding) or potential differences between the behavior of VLPs and native virus, which has been discussed amongst experts in the field (R. Atmar, personal communication). Nonetheless, in the absence of a means by which to cultivate HuNoV and/or the availability of broad strain panels, VLPs remain a valuable reagent in these types of studies.

In general, the ELASA T/N ratios were higher for GII strains than for GI strains. This is not unexpected as major capsid protein (VP1) amino acid sequences for GII strains differ from those for GI strains by over 61.4% [25], and our SELEX target (SMV) was a GII strain. Nonetheless, different VLP binding patterns for the four characterized aptamers suggest that they may bind to slightly different regions of the viral capsid. It could also be argued that the more broadly reactive aptamers bind to more highly conserved areas of the virus, such as the shell or P1 domains [26]. Of the three common motifs identified in this study, motif 2 was found in all four aptamers and this may imply a conserved interaction site with HuNoV. Secondary structures have been considered as possible putative binding epitopes on aptamers [27].

In an effort better predict aptamer binding epitopes, cluster analysis of the major capsid protein (VP1) sequences of each of the VLPs was undertaken, with aligned sequences matched with aptamers showing strong binding affinity (Figure S2). Aptamers 21 and 25 bound strongly to a group of closely related GII VLPs. Further amino acid sequence analysis demonstrated that SMV residue 406 (part of a fairly conserved area of the VPI that consists of residues 405–408) was more highly conserved (a V or K) among VLPs with stronger binding to aptamers 19 and 25. Aptamer 21 appeared to have multiple candidate epitope residues, including residue 65, for which the change to a Q from other residues (N, G, E, or A) seemed to eliminate binding. This residue is part of a sequence of amino acids that is quite conserved (NFVQAPQGEFT), which could help explain the relatively broad reactivity of aptamer 21. Additionally, the presence of an amino acid different from V or A at residue 394 was correlated with lower ELASA T/N ratios for aptamer 21. Finally, a sequence starting at residue 428 [FPGEQ(I/L)(I/L)] was also conserved among the six VLPs to which aptamer 21 bound most strongly. Further characterization of the putative aptamer binding sites will require mutagenic, structural, bioinformatics, and/or other analyses beyond the scope of this current work.

Antibodies, the most frequently used ligands for HuNoV capture and detection [28] [29] [30], tend to lack broad reactivity, meaning that subsequent assays developed with these antibodies lack analytical sensitivity [6]. Having an alternative ligand type showing broad reactivity to multiple HuNoV strains provides another tool upon which capture and detection assays may be based. Theoretically, aptamers having different specificities could be used as a polyvalent cocktail to impart a high level of inclusivity for virus capture and detection. They could also be used in combination with other ligands such as antibodies or peptides [31]. These sorts of applications have recently been proposed by others [32] and are the subject of future investigations.

Using the ELASA assay, we were able to approximate the SMV aptamer K_d_ values to be in the 100–200 nM range. This is similar to most commercial antibodies which have K_d_ values in the low µM to nM range (www.abcam.com). In binding studies specifically with Norwalk virus VLPs, monoclonal antibodies have been shown to have K_d_ values in the low nM range [33] and K_d_ values for enteric virus binding protein as applied to HuNoV VLPs was similarly in the range of 210–240 nM [34]. Although we did not perform K_d_ studies on the commercial antibody used in comparative studies with aptamer 25, the antibody and aptamer did perform equivalently in ELISA and ELASA, respectively. Some non-specific binding using a scrambled sequence of aptamer 25 was observed, and this is consistent with others [35]. However, we note that few studies use scrambled sequences to evaluate non-specific binding of nucleic acid aptamers. The degree of non-specific binding observed here may be a function of the concentration of the aptamer used in the assay, as at 1 µM we approached saturation of the signal. It could also be a feature specific to aptamer 25, and could therefore differ were the other aptamers to be screened in this manner. Future studies using a range of aptamer concentrations and screening a wider variety of VLPs and aptamers would be a logical next step.

Aptamer 25 performed quite well when applied to a virus concentrate derived from an artificially contaminated model food product using a combined virus pre-concentration followed by AMC-qPCR assay format. Use of aptamers for pre-concentration of microbes has been reported recently by others [36]. Comparatively speaking, the detection limit and capture efficiency of this method, at 10 RNA copies and 36%, respectively, were comparable to those for HuNoV immunomagnetic separation-RT-qPCR assays applied to artificially contaminated fresh produce items [29] [30] and clinical specimens [28]. Our detection limits were also similar if not better than those for other capture approaches that use more non-specific ligands such as histo-blood group antigens [37] [38] [39] and porcine gastric mucin [40].

In conclusion, we report the identification of ssDNA aptamers having binding affinity and inclusivity to various HuNoV strains. We also confirmed the performance of these aptamers in capture and detection assays for HuNoV in outbreak associated human stool suspensions and in an artificially contaminated food matrix. Consistent with recent literature [36], nucleic acid aptamers are promising ligands to facilitate pathogen capture and detection in complex sample matrices. As demonstrated in this study, aptamers may be good alternatives to HuNoV antibodies due to their relatively high inclusivity, specificity, ease of synthesis, and lower cost. Their use in detection assays is obvious, but they may also have utility in other sorts of assays, such as those that seek to discriminate between infectious and non-infectious virus particles [8]. Future work will continue to focus on these sorts of applications.

## Supporting Information

Figure S1 **Scrambled aptamer analysis.** The binding of biotinylated aptamer 25 was compared to that of a labeled scrambled aptamer (25 S) in competitive and non-competitive ELASA. For the latter, combinations of biotinylated labeled aptamer 25 with unlabelded 25 S, and vice versa, were used. Labeled aptamers were added at a concentration of 1 µM; unlabeled aptamers were used in 4-fold excess. Experiments were done by triplicate. Different letters indicate statistically significant differences between treatment groups (*p*<0.05).
(TIFF)

Figure S2 **Maximum likelihood tree for VLPs.** Full-length protein sequences of the VP1 protein of each of the VLPs tested were aligned using Clustal W alignment in the Molecular Evolutionary Genetics Analysis program (MEGA 6.0) (http://www.megasoftware.net/). Aligned sequences were matched with the aptamers that showed the strongest (+++) binding affinity to each VLP.
(TIFF)
