# Supplementary figures and images for: Selection, Characterization and Application of Nucleic Acid Aptamers for the Capture and Detection of Human Norovirus Strains
Source: PLoS One. 2014 Sep 5;9(9):e106805. doi: 10.1371/journal.pone.0106805 (PMC4156411; doi:10.1371/journal.pone.0106805)

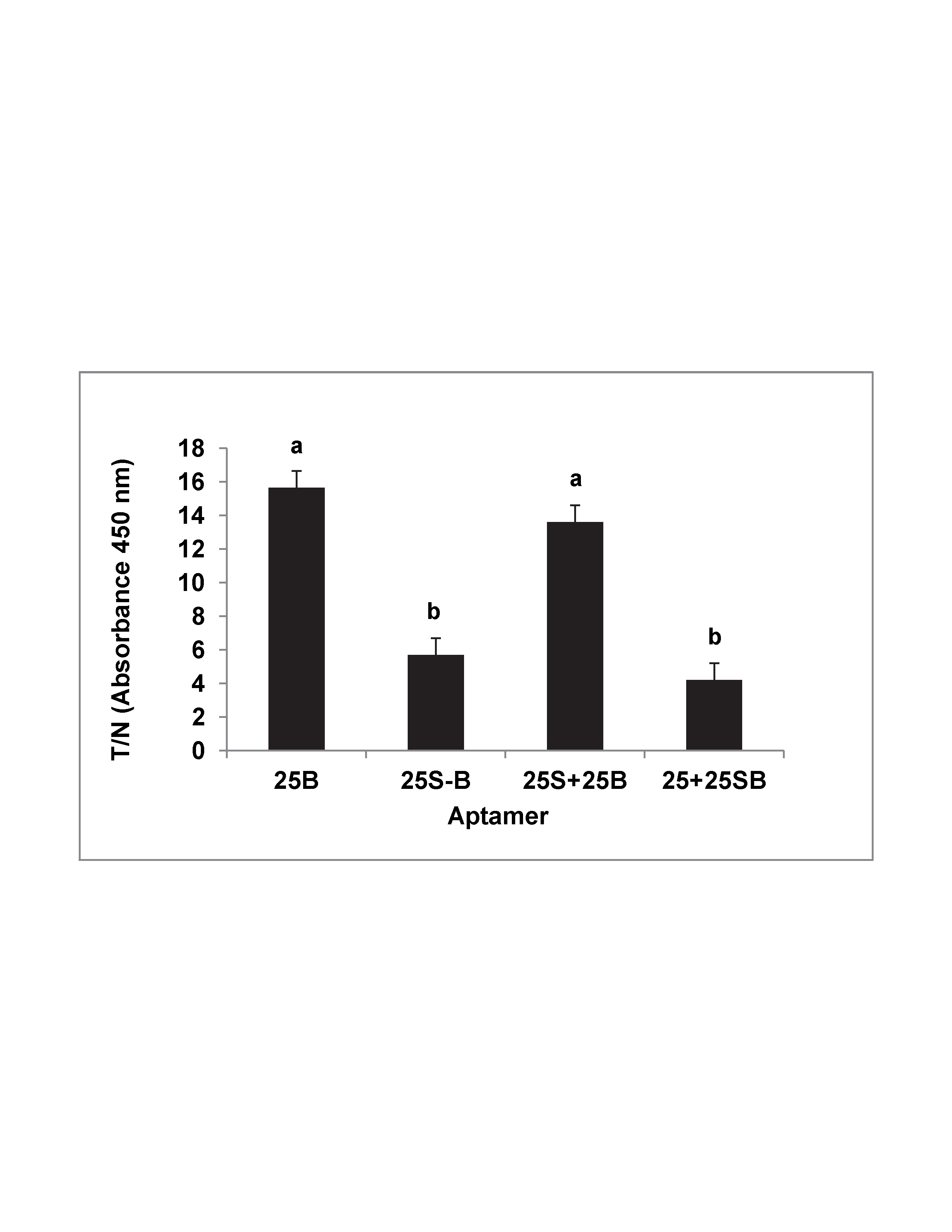

Supplement: Figure S1 — Scrambled aptamer analysis. The binding of biotinylated aptamer 25 was compared to that of a labeled scrambled aptamer (25 S) in competitive and non-competitive ELASA. For the latter, combinations of biotinylated labeled aptamer 25 with unlabelded 25 S, and vice versa, were used. Labeled aptamers were added at a concentration of 1 µM; unlabeled aptamers were used in 4-fold excess. Experiments were done by triplicate. Different letters indicate statistically significant differences between treatment groups (p<0.05). (TIFF) [file pone.0106805.s001.tiff]

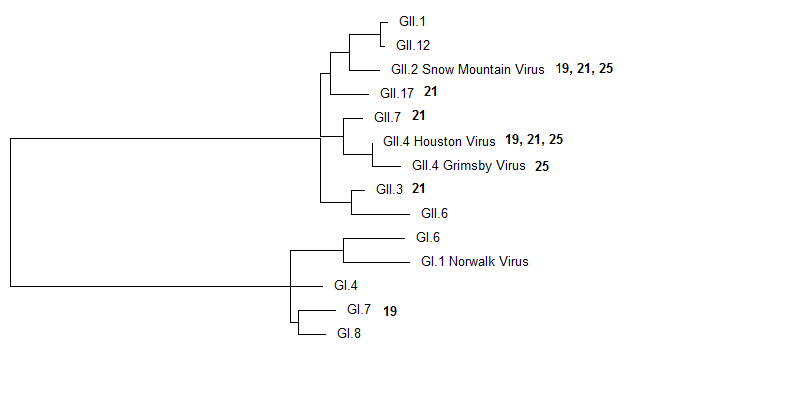

Supplement: Figure S2 — Maximum likelihood tree for VLPs. Full-length protein sequences of the VP1 protein of each of the VLPs tested were aligned using Clustal W alignment in the Molecular Evolutionary Genetics Analysis program (MEGA 6.0) (http://www.megasoftware.net/). Aligned sequences were matched with the aptamers that showed the strongest (+++) binding affinity to each VLP. (TIFF) [file pone.0106805.s002.tiff]
